# N-Terminal (1→3)-β-d-Glucan Recognition Proteins from Insects Recognize the Difference in Ultra-Structures of (1→3)-β-d-Glucan

**DOI:** 10.3390/ijms20143498

**Published:** 2019-07-16

**Authors:** Yoshiyuki Adachi, Masaki Ishii, Takashi Kanno, Junko Tetsui, Ken-ichi Ishibashi, Daisuke Yamanaka, Noriko Miura, Naohito Ohno

**Affiliations:** Laboratory for Immunopharmacology of Microbial Products, School of Pharmacy, Tokyo University of Pharmacy and Life Sciences, Tokyo 192-0392, Japan

**Keywords:** β-d-glucan, glucan binding protein, host defense, innate immunity

## Abstract

Recognition of (1→3)-β-d-glucans (BGs) by invertebrate β-1,3-d-glucan recognition protein (BGRP) plays a significant role in the activation of Toll pathway and prophenoloxidase systems in insect host defense against fungal invasion. To examine the structure diversity of BGRPs for the recognition of physiochemically different BGs, the binding specificity of BGRPs cloned from four different insects to structure different BGs was characterized using ELISA. Recombinant BGRPs expressed as Fc-fusion proteins of human IgG1 bound to the solid phase of BGs. Based on the binding specificities, the BGRPs were categorized into two groups with different ultrastructures and binding characters; one group specifically binds BGs with triple-helical conformation, while the other group recognizes BGs with disordered conformations like single-helical or partially opened triple helix. The BGRPs from the silkworm and the Indian meal moth bound to the BGs with a triple-helical structure, whereas BGRPs from the red flour beetle and yellow mealworm beetle showed no binding to triple-helical BGs, but bound to alkaline-treated BGs that have a partially opened triple-helical conformation. This evidence suggests that the insect BGRPs can distinguish between different conformations of BGs and are equipped for determining the diversity of BG structures.

## 1. Introduction

Innate immune system is ubiquitously equipped in various organisms to recognize molecular patterns on pathogens [1]. To accomplish host defense mechanisms in invertebrate, it is important to discriminate the large number of potential pathogens from itself using a restricted number of germ-line encoded receptors and binding proteins. Invertebrates have protective factors that recognize various biological components of microbes. In particular, β-glucans act on the host glucan-binding proteins to activate the prophenoloxidase system, and lead to the expression of various innate immune related genes [2]. Insects possess unique pattern-recognition receptors, called PGRP and BGRP against peptidoglycan (PG) and (1→3)-β-d-glucan (BG), respectively [3]. These recognition proteins initiate activation of pro-phenoloxidase, which leads to melanin formation in addition to Toll and Imd pathways [4]. The interaction of BGRP with BG and PGRP with PG activates serine proteases which subsequently alternate the pro-phenoloxidase to the phenoloxidase [5]. This reaction system can be applied for the detection of BG and PG using body fluid obtained from silkworm larvae [6]. However, it does not distinguish the content of BG and PG in a test sample, because the fluid contains both BGRP and PGRP [6]. 

The structural diversity of BGs has been reported. Water-soluble BGs generally possess (1→6)-β-d-glucopyranosyl branches with various frequencies and lengths on the (1→3)-β-d-glucan main chain. The ultrastructure of the branched (1→3)-β-d-glucans, such as sonifilan from *Schizophyllum commune* [7,8] and laminarin from *Laminaria digitate* [9], is the triple helix. The triple-helical conformation can be transiently converted to single-strand random coiled or single-helical form, in other words partially opened triple helix, by serial treatment with alkaline and neutralization [10]. It is hypothesized that the innate immune system is able to recognize the structural diversity of the BGs [11]. Therefore, we have isolated four kinds of BGRPs from different insects and examined the binding specificity towards structure-differed BGs in this study. As a result, we have found the two classes of BGRPs, from lepidopteran and coleopteran, bound to the triple-helical conformation and the single-strand conformation of BGs, respectively. This evidence supports that innate immune systems in insects survey different molecular patterns which occur in conformational alteration of polysaccharide.

## 2. Results

### 2.1. Evaluation of Direct Binding Activity of BGRP-Fc Proteins to Solid Phase BG by ELISA

It has been well documented that Sonifilan (SPG) and laminarin form a triple-helical ultrastructure in physiological solution [7,8]. The triple-helical conformation can be altered by alkaline treatment to random coiled, then partially opened, triple-helical conformation after neutralization with acid solution for renature [9]. Although this conformational change is reversible, the partially open triple-helical conformation remained for 35.5 h after the neutralization of the alkaline-treated laminarin [9]. Based on the experimental conditions described in previous reports [8,9], we treated SPG and laminarin with 0.5 M NaOH and neutralized them to prepare conformationally different BGs.

#### 2.1.1. Binding of BGRPs to Solid Phase of SPG

**Figure 1:**
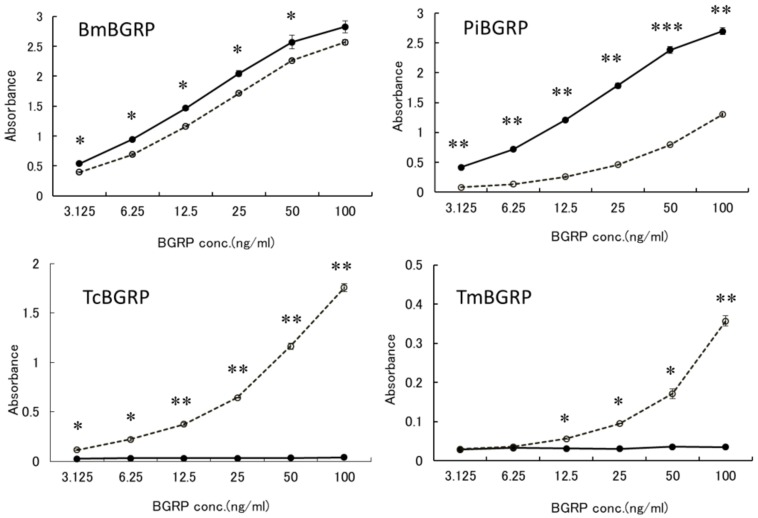
Binding activity of β-1,3-d-glucan recognition protein (BGRP)-Fc to solid-phase of (1→3)-β-d-glucans (BGs). Binding of BGRP-Fc to Sonifilan (SPG) (black circle) and alkaline-treated SPG (AT-SPG) (open circle, dashed line) were measured. SPG and AT-SPG were coated on ELISA plate at 1 µg/ml. Each sample concentration was measured by duplicate in an experiment. The data show representative results performed at least twice. Absorbance of each sample was measured in duplicate in the experiment. Data shown are representative of two independent experiments with similar tendency. Statistical significance between native BG and AT-BG on ELISA plate were found to be as follows: * *p* < 0.05, ** *p* < 0.01, *** *p* < 0.001, respectively.

To examine the binding ability of BGRP-Fc to structurally different β-glucans, Sonifilan (SPG), laminarin, and their alkaline-treated glucans, were tested by ELISA. *Bombyx mori*-derived BGRP (BmBGRP) and *Plodia interpunctera*-derived BGRP (PiBGRP) showed significant binding to both SPG and alkaline-treated SPG (AT-SPG) (Figure 1 upper). 

There was no difference in the biding ability of BmBGRP to SPG and AT-SPG. However, PiBGRP showed higher binding to SPG than AT-SPG. In contrast, *Tribolium castaneum*-derived BGRP (TcBGRP) and *Tenebrio molita*-derived BGRP (TmBGRP) showed no binding to SPG but bound to AT-SPG (Figure 1 lower). 

#### 2.1.2. Binding of BGRPs to Solid Phase of Laminarin

**Figure 2:**
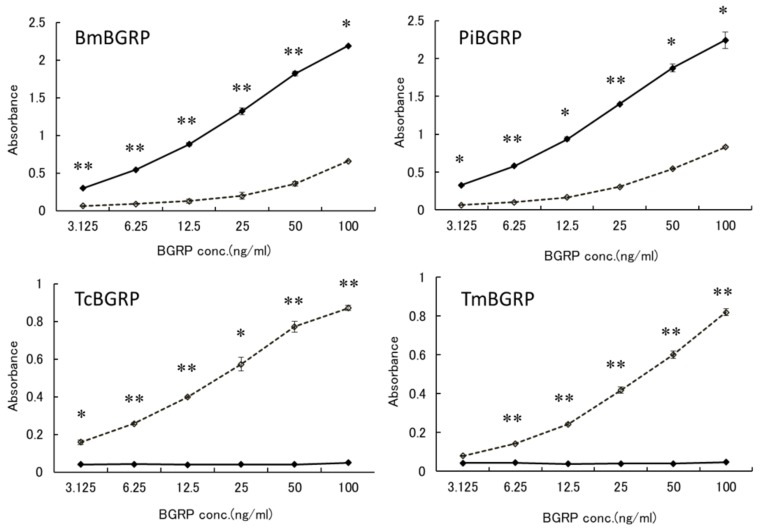
Binding activity of BGRP-Fc to solid-phase of BGs. Binding of BGRP-Fc to laminarin (black diamond) and AT-laminarin (open diamond, dashed line) measured by ELISA. Laminarin and AT-laminarin were coated on ELISA plate at 10 µg/mL. Absorbance of each sample was measured in duplicate in the experiment. Data shown are representative of two independent experiments with similar tendency. Statistical significance between native BG and AT-BG on ELISA plate were found to be * *p* < 0.05 and ** *p* < 0.01, respectively.

To confirm that these specificities might be resulted from the conformational difference of BG, anoother BG, laminarin, which has lower MW and lower branching ratio of 1, 6-β-monoglucoside than SPG, was applied to the binding assay. BmBGRP and PiBGRP bound well to undenatured laminarin (Figure 2 upper). TcBGRP and TmBGRP showed no binding to laminarin, but significantly bound to AT-laminarin as well as AT-SPG (Figure 2 lower). 

### 2.2. Competitive Effect of Liquid Phase BGs in ELISA

We have reexamined the binding specificity of these BGRPs to liquid phase of BGs as competitors against solid phase of conformationally different BGs. 

#### 2.2.1. Binding Difference of BGRPs to the Different Conformation of SPG in Liquid Phase 

**Figure 3:**
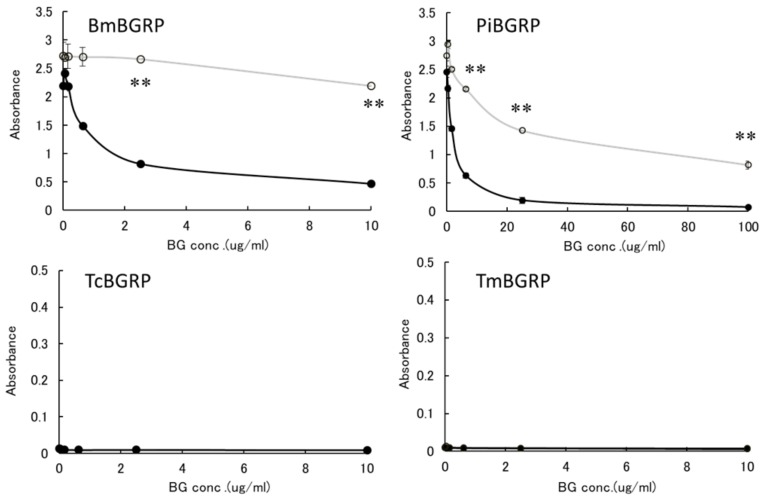
Binding activity of BGRP-Fc to SPG and AT-SPG in the liquid phase. Competition with liquid phase of SPG (black circle) and AT-SPG (open circle, gray line) in the BGRP-Fc (100 ng/mL) binding to SPG (10 µg/mL). *Tribolium castaneum*-derived BGRP (TcBGRP)-Fc and *Tenebrio molita*-derived BGRP (TmBGRP)-Fc failed to bind solid phase of SPG. Absorbance of each sample was measured in duplicate in the experiment. Data shown are representative of two independent experiments with similar tendency. Statistical significance between native BG and AT-BG in the liquid phase was shown as ** *p* < 0.01.

**Figure 4:**
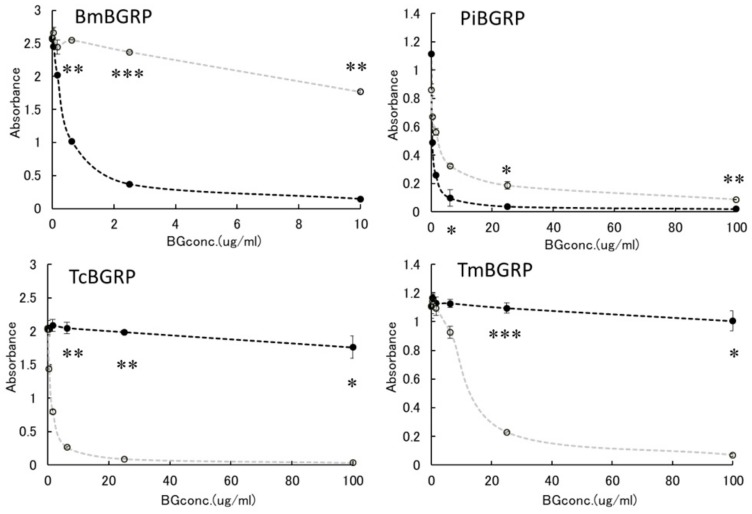
Binding activity of BGRP-Fc to SPG and AT-SPG in the liquid phase. Competition with liquid phase of SPG (black circle, dashed line) and AT-SPG (open circle, dashed gray line) in the BGRP-Fc (100 ng/mL) binding to AT-SPG (10 µg/mL). Absorbance of each sample was measured in duplicate in the experiment. Data shown are representative of two independent experiments with similar tendency. Statistical significance between native BG and AT-BG in liquid phase was shown as * *p* < 0.05, ** *p* < 0.01, *** *p* < 0.001.

First, the effect of liquid phase of SPG and AT-SPG on BGRPs binding to SPG coated on ELISA plate was examined. As shown in Figure 3 and Figure 4, SPG or AT-SPG was immobilized on an ELISA plate. In the liquid phase, SPG and AT-SPG competitively inhibited the binding of BmBGRP and PiBGRP to solid phase SPG. In the case of BmBGRP and PiBGRP, binding to solid phase SPG was strongly inhibited by SPG (Figure 3 upper). The binding of BmBGRP to AT-SPG was not strongly inhibited by AT-SPG in the liquid phase, but strongly inhibited by SPG (Figure 4 upper). If it is assumed that BmBGRP binds to BG with a triple-helical conformation, the triplex portion remained in solid phase AT-SPG, and BmBGRP may bind to partially existing triple-helical conformation in the AT-SPG. It was suggested that SPG in the liquid phase acted as an inhibitor for the binding of BmBGRP and PiBGRP. 

On the other hand, TcBGRP and TmBGRP did not bind to the solid phase SPG, and the competitive reaction could not be observed (Figure 3 lower). However, the binding properties on the AT-SPG coated plate were observed well. The absorbance decreased with increasing concentration of AT-SPG (Figure 4 lower). From these results, it was shown that BmBGRP and PiBGRP tend to bind to SPG, and TcBGRP and TmBGRP tend to bind to AT-SPG.

#### 2.2.2. Binding Difference of BGRPs to the Different Conformation of Laminarin in Liquid Phase 

**Figure 5:**
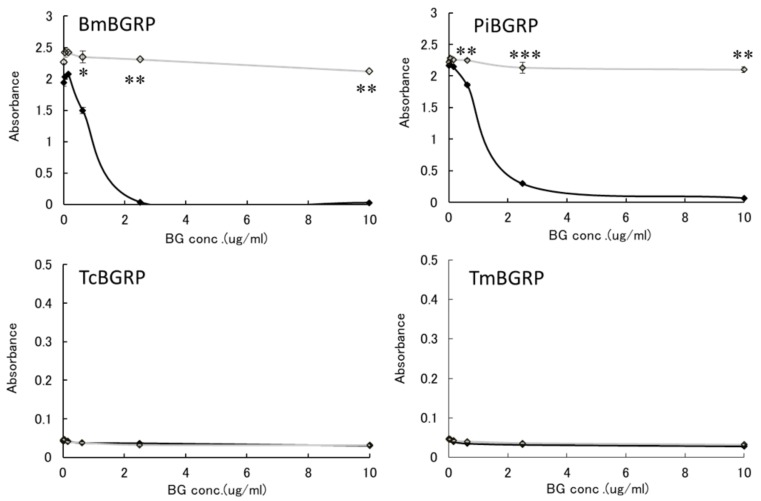
Binding activity of BGRP-Fc to laminarin and AT-laminarin in the liquid phase. Competition with liquid phase of laminarin (black diamond) and AT-laminarin (open diamond, gray line) in the BGRP-Fc binding to solid phase of laminarin (10 µg/mL). TcBGRP-Fc and TmBGRP-Fc failed to bind solid phase of laminarin (10 µg/mL). Absorbance of each sample was measured in duplicate in the experiment. Data shown are representative of two independent experiments with similar tendency. Statistical significance between native BG and AT-BG in liquid phase was shown as * *p* < 0.05, ** *p* < 0.01, *** *p* < 0.001.

**Figure 6:**
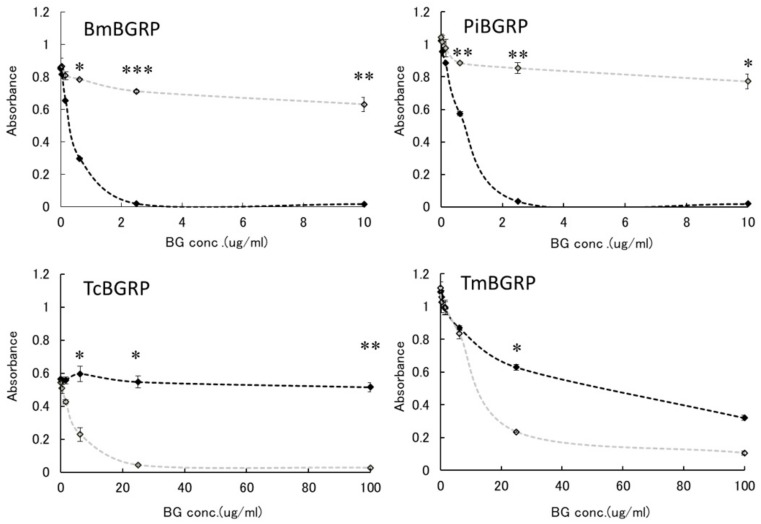
Binding activity of BGRP-Fc to laminarin and AT-laminarin in the liquid phase. Competition with liquid phase of laminarin (black diamond, dashed line) and AT-laminarin (open diamond, dashed gray line) in the BGRP-Fc binding to solid phase of AT-laminarin (10 µg/mL). Absorbance of each sample was measured in duplicate in the experiment. Data shown are representative of two independent experiments with similar tendency. Statistical significance between native BG and AT-BG in liquid phase was shown as * *p* < 0.05, ** *p* < 0.01, *** *p* < 0.001.

Similar results were observed in the competition assay using laminarin and AT-laminarin. (Figure 5). Particularly, in case of TcBGRP and TmBGRP, the binding inhibition with AT-laminarin in liquid phase was observed in the binding against AT-laminarin (Figure 6). 

Above results suggest the binding of BmBGRP and PiBGRP tend to be high on triple-helical BGs, although TcBGRP and TmBGRP have higher binding ability to AT-SPG and AT-laminarin, which form partially opened triple helical conformation.

### 2.3. Binding Kinetics of BGRPs to SPG and AT-SPG

**Figure 7:**
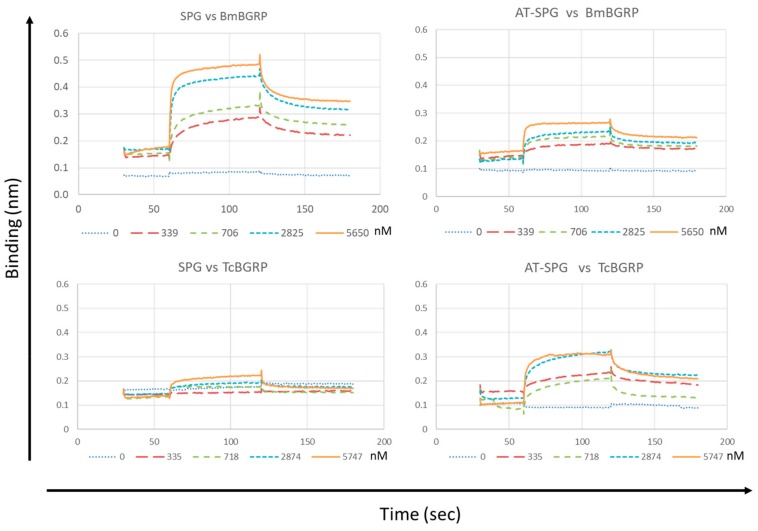
Binding affinity measurements of BmBGRP and TcBGRP to SPG or AT-SPG on BG-conjugated biosensors. KD, Ka, and Kd were measured and calculated by the BLitz system. All experiments were performed with at least two independent occasions in duplicate readings. The X-and Y-axis depicts the time in seconds and the binding in nm, respectively.

The interaction of BmBGRP and TcBGRP with different conformers of SPG was recapitulated and quantified using biolayer interferometry (BLitz). As shown in Figure 7, the base line of the sensor tip without the analyte was around 0.1 after loading with Biotin-conjugated SPG- or AT-SPG on the Streptavidin-coated surface. The control line was monitored by running with PBS containing 0.5% BSA. A concentration-dependent increase in the binding of the BmBGRP with SPG was observed. In contrast, interaction of TcBGRP with SPG-loaded sensor tip was quite lower than BmBGRP. However, the binding of TcBGRP was significantly higher than BmBGRP in the case of interaction with AT-SPG-loaded sensor tip (Figure 7). The affinity (KD) of BmBGRP toward SPG and AT-SPG was calculated to be 0.29 and 0.20 µM respectively. Rmax of BmBGRP to SPG and AT-SPG was 0.26 and 0.07, respectively (Table 1), suggesting binding site for BmBGRP on SPG was reduced by alkaline-treatment. However, the KD of TcBGRP toward SPG and AT-SPG was 1.77 and 0.71 µM respectively, suggesting affinity of TcBGRP was improved by alkaline-treatment of SPG. These results suggest that TcBGRP tends to interact with alkaline-denatured conformation of BG. 

## 3. Discussion

Although insects or invertebrate animals do not have the acquired immunity to the pathogens, they are able to recognize the molecules on the pathogenic microorganisms by using pattern recognition receptor molecules [1]. BG-binding proteins and BGRP are well-known pattern recognition receptor molecules as applied in the Limulus amebocyte lysate (LAL) assay and silkworm larvae plasma (SLP) reagent set (Wako Pure Chemical Industries, Ltd.) for detection of (1→3)-β-d-glucan [6,12]. Among the recognition receptor proteins, BGRPs are well characterized molecules as their 3-D structure are clarified by X-ray crystallography and NMR [13,14]. In this study, we have isolated four kinds of BGRPs from different insects and examined the binding specificity towards structure-differed BGs.

BmBGRP and PiBGRP showed consistent higher binding to native BGs which have helix conformation. This binding specificity was confirmed by reverse experiment using competitor BGs in liquid phase prior to interacting with solid-phase BGs on ELISA plate. Binding ability of BmBGRP and PiBGRP to alkaline-treated BG were also observed either in solid phase or liquid phase. Even the alkaline-treated BGs partially possess helical conformation in the solution [11]. BmBGRP and PiBGRP may bind to the scattering helical portion remaining in the AT-SPG and AT-laminarin. In contrast, TmBGRP and TcBGRP had no binding ability to native BGs which have triple-helical conformation. In the case of binding of TcBGRP and TmBGRP, these proteins bound to alkaline-treated BGs, but not to native BGs even in solution and solid-phase. It was suggested that the Fc portion of those BGRPs does not bind nonspecifically to BGs immobilized on the ELISA plate. Compared to TcBGRP, TmBGRP seems to have a lower binding affinity for AT-SPG. The reason for this lower affinity of TmBGRP towards AT-SPG is not clear. However, on solid phase, the binding pattern of TmBGRP to AT-SPG was consistent to that observed on liquid phase. As shown in Figure 1 and Figure 4, the maximum absorbance was 0.35 with 1 µg/mL of AT-SPG and 1.1 with 10 µg/mL of AT-SPG. The difference in the glucose branching ratio in the side chain between SPG and laminarin may affect the corresponding reactivity to TmBGRP. Laminarin has one 1,6-linked branched glucose for every seven glucose residues on the main 1,3-beta-glycosidic linkage [9]. However, SPG had one 1,6-linked side branch glucose for every third glucose on the main 1,3-beta-glycosidic linkage [6]. Many side chain glucose residues on AT-SPG may interfere with the interaction of TmBGRP with 1,3-glucan main chain.

It was reported that alkaline-treatment would form partially opened triple helical conformation in BG [9,11,15]. Based on prior reports [8,9,11,15,16], we applied the alkaline-treatment to SPG and laminarin, which exist in a triple helical conformation in neutral conditions. TmBGRP and TcBGRP did not show any reactivity to native SPG and laminarin, but were reactive to alkaline-treated BGs. These results strongly suggest that TmBGRP and TcBGRP failed to recognize tightly spiraled glucosyl-linkage in the 1,3-β-d-glucan strands.

The conversion between helix and random coiled conformers can be mediated by different chemical or physical treatments [15]. Treatment of the helix SPG with NaOH has been used to prepare disordered forms [15,17]. Aketagawa et al. [18] suggested that treatment of SPG with NaOH alters the triple-helix to single chains [17]. This mechanism implies that immediately after treatment with NaOH the molecular weight should be one-third of the untreated glucan, however, experimental evidence has shown that denatured SPG has the same molecular weight as untreated SPG [18]. An alternative explanation that is consistent with the observations regarding molecular weight would be that NaOH treatment results in a partially disordered the helix rather than completed strand separation. 

It was reported that for glucans with different conformation but the same degree of polymerization, the triple-helix is 100 to 1000 times less potent than the single-helix in activation of limulus coagulation including factor G [18]. It was speculated that LAL activity would be dependent on the degree of partial opening of the triple-helix after NaOH treatment. More stable conformers at different degrees of strand opening with aniline blue and analyzed their relationship to LAL activation [11]. These studies suggested, for both a low molecular weight and high molecular weight glucan, that conformations with a higher degree of partial opening (single helix structure) were more effective in activating the LAL assay. It was demonstrated that there might be a gradient of activity between conformers with a greater degree of opening and the triple-helix forms.

A report revealed that the ligand BG structure co-crystalized with PiBGRP was triple-helical conformation [13]. This evidence consistent with the present study that PiBGRP preferentially binds to triple helical conformation of BGs. Another report also supports the conformational dependency of BmBGRP, because silkworm (*Bombyx mori*) larvae fluid showed higher sensitivity to the triple helical BGs than alkaline-treated BGs in the melanin formation triggered by BG and BGRP interaction [16].

In the Surface plasmon resonance (SPR) analysis, the NaOH-treated laminarin showed impaired affinity to β-GRP N-terminal protein from *Bombyx mori* [14], indicating that β-GRP N-terminal portion binds the triple-helical structure of (1→3)-β-d-glucan. They studied the time-dependent recovery of the binding affinity of NaOH-treated laminaran to β-GRP after neutralization and confirmed that β-GRP preferably binds the refolded triple helical structure of laminarin [14]. 

In the present study, the binding specificity of BGRPs from Bm and Pi was similar to the former study examined using with β-GRP N-terminal protein from *Bombyx mori* [14]. However, quite different specificity was observed by TcBGRP and TmBGRP in this investigation. The TcBGRP and TmBGRP showed less binding to native form of laminarin and SPG, and preferentially bind to NaOH-treated laminarin and SPG possessing partially opened triple-helical conformation. In the BLitz analysis, the binding kinetics of the BmBGRP and TcBGRP were examined by pre-loading SPG- or AT-SPG to the biolayer interferometry sensor. The KD of BmBGRP toward SPG and AT-SPG was not changed, but Rmax of BmBGRP to SPG was reduced from 0.26 to 0.07 in the interaction with AT-SPG (Table 1), suggesting binding site for BmBGRP on SPG was reduced by alkaline treatment. On the contrary, the KD of TcBGRP toward SPG and AT-SPG was 1.77 and 0.71 µM respectively, and Rmax of TcBGRP to SPG and AT-SPG was increased from 0.09 to 0.18. These results suggest that NaOH-treatment of triple-helical BG resulted in conformational change, which is a good target for TcBGRP. 

A report revealed that the ligand BG structure co-crystalized with PiBGRP was triple-helical conformation [13]. This evidence is consistent with the present study that PiBGRP preferentially binds to triple helical conformation of BGs. Another report also supports the conformational dependency of BmBGRP, because silkworm (*Bombyx mori*) larvae fluid showed higher sensitivity to the triple-helical BGs than alkaline-treated BGs in the melanin formation triggered by BG and BGRP interaction [16]. Mammalian (1→3)-β-d-glucan receptor protein, Dectin-1, also binds the helical conformation of BGs [19]. 

**Figure 8:**
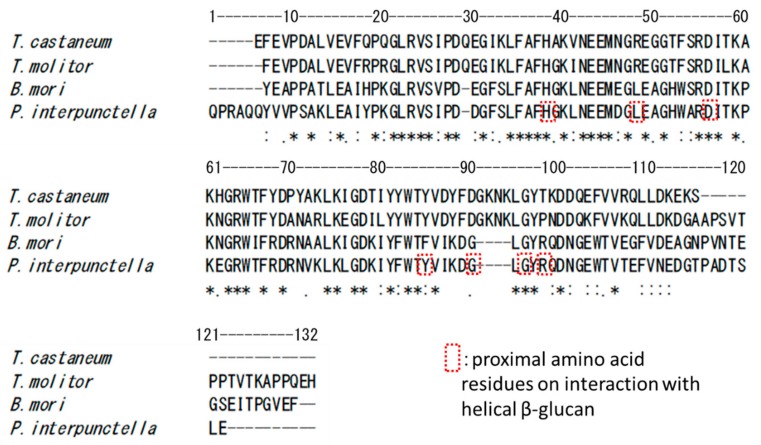
Sequence alignment of N-terminal domains of BGRP from *Plodia interpunctella* (*P. interpunctella*), *Bombyx mori* (*B. mori*), *Tenebrio molitor* (*T. molitor*), and *Tribolium castaneum* (*T. castaneum*). The amino acid residues surrounded by a dashed square are amino acid residues proximal to the helical β-glucan [13].

From the results of amino acid sequence alignment of the four BGRPs tested, the obvious different amino acid residues are seven residues consisted of -DYFDGKNK- in TcBGRP and TmBGRP, while PiBGRP and BmBGRP have -IKDG- instead. It is reported that the amino acid residues of Y78, G83, G85, and R87 on PiBGRP interact with the glucose residues of triple-helical (1→3)-β-d-glucan [13]. These amino acid residues reside around -IKDG- on PiBGRP and BmBGRP, while TcBGRP [20] and TmBGRP [21] have -DYFDGKNK- (Figure 8). The binding specificity of TcBGRP and TmBGRP to opened helical glucans might be correlated with the stretched peptide portion sandwiched with Y78 and G85 of PiBGRP corresponding to Y85 and G96 of TcBGRP and TmBGRP (Figure 8). 

Invertebrates utilize various binding molecules for (1→3)-β-d-glucans [2]. The preferential higher reactivity to NaOH-treated (1→3)-β-d-glucan is demonstrated in the innate immune system in horseshoe crab [17]. The LAL requires alkaline-treatment of test samples before measuring water soluble (1→3)-β-d-glucans. LAL is less reactive to triple-helical BG [22]. Therefore, it is likely that innate immune system against (1→3)-β-d-glucan may have diverse recognition in response to conformationally different polysaccharides to accomplish host defense to various microorganisms.

## 4. Materials and Methods 

### 4.1. Insect larvae

*Bombyx mori* (Kinsho strain) and *Tenebrio molitor* were purchased from Kogensha Co., Ltd (Nagano, Japan) and Asahi Pet (Yokohama, Japan), respectively. *Plodia interpunctera*, and *Tribolium castaneum* were kindly supplied by Dr. Akihiro Miyanoshita and Dr. Taro Imamura, National Food Research Institute (Tsukuba, Japan). 

### 4.2. β-Glucans

Sonifilan (Schizophyllan, SPG) and laminarin from *Laminaria digitata* were purchased from Kaken Pharmaceutical Co., Ltd (Tokyo, Japan) and Sigma-Aldrich (St. Louis, MO, USA). The laminarin solution was prepared by solubilized in pyrogen-free distilled water at 10 mg/mL. For alkaline treatment, the β-glucan neutral solutions, SPG and laminarin, were mixed with equal volume of 1 M NaOH, then neutralized in diluting with 0.1 M Tris-HCl buffer (pH 8.0) to prepare 1 mg/mL of alkaline-treated β-glucans, referred as AT-SPG and AT-laminarin, respectively. Biotinylated SPG (Biotin-SPG) was prepared as described in [23].

### 4.3. Preparation of BGRP-Fc Molecules

We prepared recombinant carbohydrate recognition domain of BGRP conjugated with human IgG Fc protein. The various BGRP cDNA from *B. mori* [24], *T. molitor* [21], *P. interpunctera* [13], and *T. castaneum* [20] was amplified by PCR using KOD polymerase and specific oligonucleotide primers listed in Table 2. These cDNAs were inserted into pDisplay vector (Invitrogen), which was already ligated with human IgG1 Fc cDNA. The expression vectors were transduced into 293T cells by using the Lipofectamine LTX (Invitrogen, Thermo Scientific, Waltham, MA, USA). BGRP-Fc proteins were isolated from a culture supernatant of 293T cells. The protein concentration of the recombinant BGRP-Fc proteins were determined by sandwich ELISA using anti-human IgG-Fc (Jackson Laboratory, West Grove, PA, USA), horse radish peroxidase (HRP)-conjugated anti-Hemagglutinin -tag monoclonal antibody (Santa Cruz, Dallas, TX, USA), and purified soluble dectin-1-Fc proteins possessing Hemagglutinin-tag, as a capture antibody, a detection antibody, and standard protein, respectively [25].

### 4.4. Preparation of BGRP-CRD

Hexahistidine-tagged carbohydrate recognition domains of BGRPs were prepared using cold shock-promoted protein expression by *E. coli* BL21. The PCR products of N-terminal portion of BGRP were inserted into multiple cloning sites of pCold-I (TaKaRa Bio Inc., Shiga, Japan). This vector is capable of expressing a target protein at low temperature (15 °C) using a cold shock promoter cspA. It was constructed by insertion of BGRP cDNAs into multiple cloning sites of a cold shock vector pCold-I (TaKaRa Bio Inc., Japan). The construct was composed of the (His)_6_-tag and BGRP. For the expression of *Bombyx mori* BGRP, a DNA fragment encoding Tyr1-Phe119 was cloned into pCold-I vector. For *P. interpunctella* (Gln1-Glu117), *T. castaneum* (Glu1-Ser110), and *T. molitor* (Phe1-His126), each expression plasmid was transformed into the *E. coli* strain BL21 (DE3). The transformed cells were grown in LB medium at 37 °C and induced with 0.1 mM isopropyl β-d-thiogalactoside (Wako) for 24 h at 15 °C. The harvested cells were suspended in a buffer containing 50 mM Tris-HCl (pH 8.0), 50 mM NaCl, and sonicated. After centrifugation, the supernatants were collected and applied to a Co Sepharose column (Clontech, Mountain View, CA, USA) equilibrated with PBS (8 mM Na_2_HPO_4_, 1 mM KH_2_PO_4_, 137 mM NaCl and 3 mM KCl, pH 7.4). After washing the column with PBS, the proteins were eluted with PBS containing 500 mM imidazole. After dialysis against PBS containing 3 mM reduced glutathione and 0.3 mM oxidized glutathione, the fused proteins were dialyzed with PBS.

### 4.5. Binding Assay of BGRP-Fc to β-Glucans by ELISA

SPG and AT-SPG, and laminarin and AT-laminarin were diluted with Tris-HCl buffered saline (pH 8.0) at 1 and 10 μg/mL, then coated on a 96-well ELISA plate (Nunc maxisorp plate, Thermo Scientific, Waltham, MA, USA) by incubating overnight at 4 °C. The unbound excess β-glucans were washed off with PBS containing 0.05% Tween 20 (PBST), and the plate was covered with PBS containing 0.5% BSA (BPBS) for 2 h at room temperature. After blocking, various concentrations of BGRP-Fc proteins (0 to 100 ng/mL) were added on each well and incubated for 1 h at room temperature. The bound BGRP-Fcs were detected with anti-human IgG Fc antibody conjugated with HRP, TMB substrate solution (Kirkegaard & Perry Laboratories, Inc., Gaithersburg, MD, USA), and 1 M Phosphate. Absorbance at 450 nm after subtraction of OD630 was measured by microplate reader (Corona MTP-450, Tokyo, Japan). Absorbance of each sample was measured in duplicate in the experiment. The data shown are representative of the experiment conducted at least twice. The significance of the differences between the means was assessed by the Student’s *t*-test. 

### 4.6. Competitive ELISA Using Liquid Phase of β-Glucans

SPG and AT-SPG, and laminarin and AT-laminarin were diluted with Tris-HCl buffered saline (pH 8.0) at 10 μg/mL, then coated on a 96-well ELISA plate (Nunc maxisorp plate, Thermo Scientific) by incubating overnight at 4 °C. The unbound excess β-glucans were washed off with PBST, and the plate was covered with PBS containing BPBS for 2 h at room temperature. In parallel, the β-glucans solution (0 to 100 μg/) in BPBS were mixed with each BGRP-Fc for 1 h at room temperature. The BGRP-Fc and β-glucan solution was added to the ELISA plate precoated with various β-glucans. The bound BGRP-Fcs to the solid phase of β-glucans were probed with anti-human IgG Fc antibody conjugated with HRP. The enzyme activity was monitored by adding TMB substrate and 1 M phosphate. Absorbance at 450 nm after subtraction of OD630 was measured by micro plate reader (Corona MTP-450, Tokyo, Japan). Absorbance of each sample was measured in duplicate in the experiment. The data shown are representative of the experiment conducted at least twice. The significance of the differences between the means was assessed by the Student’s *t*-test.

### 4.7. Binding Affinity Studies

Measurements of the association and dissociation rates of the BGRP were carried out using the direct binding of BGRPs to SPG-conjugated biosensors. The sensor chip was prepared by loading Biotin-SPG on the Streptavidin-biosensor (Fortebio, Cat no. 18-5095). AT-SPG biosensor was prepared by loading alkaline-treated and neutralized with 0.1 M Tris-HCl buffer (pH 8.0) of Biotin-SPG. Ligation of Biotin-SPG to the Streptavidin-biosensor was monitored. All readings (KD, Ka and Kd) were generated using Blitz system, and binding graphs were re-plotted using Microsoft Excel 2010. KD was calculated automatically by the software where KD = Kd / Ka. Statistical error for Ka and Kd were calculated by the software based on the replicate experiments. As the KD readings are calculated, they do not have statistical error. The data shown are representative of the experiment conducted at least twice.

## 5. Conclusions

BGRPs from insects have at least two types of specificity to the conformationally different (1→3)-β-d-glucans. BGRPs from Lepidoptera, BmBGRP, and Pi tend to interact with triple-helical conformation. On the contrary, BGRPs from Coleoptera, TmBGRP, and TcBGRP preferentially bind to alkaline-denatured ultrastructure. These results suggest that insect BGRPs can distinguish between ultrastructural changes in (1→3)-β-d-glucans. 

## Figures and Tables

**Table 1:** Binding kinetics of BGRPs to SPG- or AT-SPG.

β-Glucan	BGRP	Conc. (nM)	KD (µM)	K_a_ (1/Ms)	K_a_ Error	K_d_ (1/s)	K_d_ Error	Rmax	R Equilibrium
SPG	Bm	5650	0.29	2.09 × 10^5^	1.26 × 10^4^	6.16 × 10^−2^	6.16 × 10^−^^2^	0.2626	0.2496
	Tc	5747	1.77	7.28 × 10^4^	1.42 × 10^4^	1.29 × 10^−^^1^	1.29 × 10^−3^	0.09497	0.07264
AT-SPG	Bm	5650	0.20	3.40 × 10^5^	3.59 × 10^4^	6.79 × 10^−^^2^	6.79 × 10^−2^	0.07618	0.07358
	Tc	5747	0.71	6.63 × 10^4^	4.97 × 10^3^	4.71 × 10^−^^2^	4.71 × 10^−^^2^	0.1853	0.1649

Binding of BmBGRP and TcBGRP to sensor chip conjugated with SPG or AT-SPG was monitored by Bio-Layer Interferometry method. The binding of various concentration of BGRPs, from 339 nM to 5.7 µM, and dissociation of BGRP from the SPG or AT-SPG was analyzed for 90 seconds in each period. The KD was calculated by binding/dissociation kinetics at every concentration of BGRPs as shown in Figure 7. The Fitting-Global model using every concentration of analyte for association and dissociation kinetics was applied. The Fitting 1:1 model fits one BGRP analyte in solution binding to one binding site on the β-glucan surface. The data shown are representative of the experiment conducted at least twice.

**Table 2:** PCR primers for cDNA insert to Fc-fusion expression vector plasmids.

BGRP CRDs	Primers	Sequence
Bm	Forward	5′-CCAGATCTTACGAGGCACCACCGGCCAC-3′
	Reverse	5′-GCGGATCCGAATTCTACTCCTGGTGTTAT-3′
Pi	Forward	5′-GAGGATCCCAGCCGCGTGCGCAGCAGTAC-3′
	Reverse	5′-GACCTGCAGCCCTCGAGACTCGTGTCAGCCGG-3′
Tc	Forward	5′-GCGGATCCGAGTTTGAAGTTCCGGATGCT-3′
	Reverse	5′-GACCTCGAGCTAGACTTTTCTTTGTCTAGTAA-3′
Tm	Forward	5′-GCCAGATCTTTTGAGGTGCCAGATGCTTTG-3′
	Reverse	5′-GCCGGATCCGTGTTCTTGCGGTGGAGCCTT-3′

## Abbreviations

BG	(1→3)-β-d-glucan
BGRP	(1→3)-β-d-glucan recognition protein
PG	peptidoglycan
PGRP	peptidoglycan recognition protein
SPG	schizophylan, sonfilan
AT	Alkaline-treated
Bm	*Bombyx mori*
Pi	*Plodia interpunctera*
Tc	*Tribolium castaneum*
Tm	*Tenebrio molita*
Blitz	biolayer interferometry
LAL	*Limulus* amebocyte lysate
SLP	silkworm larvae plasma
HRP	horse radish peroxidase
TMB	3,3′,5,5′-tetramethyl-benzidene
PBS	phosphate-buffered saline
BPBS	PBS containing 0.5% bovine serum albumin
PBST	PBS containing 0.05% Tween 20
